# Longitudinal correlates of fruit and vegetable consumption with depressive symptoms among middle-aged and older adults in South Africa

**DOI:** 10.1186/s40359-022-00960-z

**Published:** 2022-11-02

**Authors:** Supa Pengpid, Karl Peltzer

**Affiliations:** 1 Department of Health Education and Behavioral Sciences, Faculty of Public Health, Mahidol University, Bangkok, Thailand; 2 Department of Public Health, Sefako Makgatho Health Sciences University, Pretoria, South Africa; 3 Department of Healthcare Administration, College of Medical and Health Science, Asia University, Taichung, Taiwan; 4 Department of Psychology, University of the Free State, Bloemfontein, South Africa; 5 Department of Psychology, College of Medical and Health Science, Asia University, Wufeng, 41354 Taichung, Taiwan

**Keywords:** Fruits, Vegetables, Incident depression, Persistent depression, Longitudinal study, South Africa

## Abstract

**Background:**

Fruit and vegetable intake may influence mental well-being. The aim of this study was to assess longitudinal associations between fruit and vegetable intake and depressive symptoms among rural South Africans.

**Methods:**

This longitudinal community study enrolled 3,891 adults (≥ 40 years) from the “Health and Ageing in Africa: A Longitudinal Study of an INDEPTH Community in South Africa (HAALSI)”. Fruit and vegetable intake was assessed by self-report at wave 1, and depressive symptoms were assessed using the Center for Epidemiological Studies Depression Scale at wave 1 and 2. Outcomes were incident and persistent depressive symptoms at wave 2. Logistic regression was used to assess the associations between fruit and vegetable intake at wave 1 and incident, and persistent depressive symptoms.

**Results:**

Results indicate that in the fully adjusted model for individuals with no depressive symptoms at baseline, we found no significant association between frequency of fruit or vegetables intake and incident depressive symptoms. We also found no significant association between frequency of fruit or vegetable intake and persistent depressive symptoms.

**Conclusion:**

Fruit and vegetable intake was not significantly associated with incident and persistent depressive symptoms.

## Background

Major depressive disorders are a major public health burden globally, including in African countries [1]. In South Africa, one out of ten people had lifetime major depression [2], and one in three older adults had depressive symptoms [3]. Relative to younger populations, older adults may experience greater negative impacts of depression on quality of life, morbidity and mortality [4]. Suggestions have been made to prevent the development of depressive disorders in the ageing community to ameliorate the depression burden among older adults [5, 6]. One possible influencing factor contributing to the onset of depressive symptoms may be poor diet, such as inadequate vegetable and fruit consumption. Fruit and vegetable intake may enhance oxidative stress or defective antioxidant defenses that are related to depression, and it has been suggested that increased intake of fruit and vegetables can be a strategy to increase brain functioning and reduce depression [7].

In four longitudinal studies among middle-aged and older adults in Australia, Taiwan and USA, higher fruit and vegetable intakes decreased the odds of incident depression [8]. In a longitudinal study among older adults in Korea, fruit intake in a dose–response manner decreased incident depressive symptoms, while vegetable intake was not significantly associated with depression status [9]. In a prospective cohort study among middle-aged and older adults (35–65 years) in Thailand, vegetable and/or fruit consumption did not significantly reduce the development of major depression [10], and in a longitudinal study among older adults in Taiwan higher fruit and vegetable consumption did not significantly decrease incident depression [11], and higher vegetable but not fruit intake decreased incident depression [12]. In a longitudinal study among middle-aged women in Australia higher fruit but not vegetable intake decreased incident depression [13].

In a further review among adults of 10 studies (6 cross-sectional and 4 longitudinal, 7 from high-income and three from middle-income countries, including China, Columbia and Malaysia) found that in four studies higher fruit intake was associated with lower odds of depression, while six studies did not find a significant association, and among eight studies, three showed that high vegetable intake decreased depression, while five studies did not find significant associations [14]. For example, a longitudinal study among adults in Canada found that after adjusting for major confounders such as physical activity and body mass index, fruit/ vegetable intake was no longer associated with incident depression [15], and in a cohort study in the UK fruit and vegetable intake decreased the risk of recurrent depressive symptoms among women but not in men [7]. In a recent review of 12 cohort studies among young people and young adults in high-income countries, most studies showed that fruit consumption decreased depression, while effects of vegetable, and fruit and vegetable intake on depression were inconsistent [17].

Most cohort studies reviewed in the general adult and older adult population were conducted in high-income countries and none in Africa, which limits generalizability of findings to low- and middle-income countries, in particular in Africa, and it remains unclear if found associations hold in populations in Africa. Therefore, the aim of this study was to assess longitudinal associations between fruit and vegetable intake and depressive symptoms among an ageing population in South Africa.

## Methods

### Sample and procedure

We analysed data from a longitudinal study (2 waves), “Health and Ageing in Africa: A Longitudinal Study of an INDEPTH Community in South Africa (HAALSI)” in Agincourt, South Africa. Sampling details have been published [9]. The first survey “(November 2014 to November 2015) included 5,059 individuals (≥ 40 years), with a response rate of 85.9%” [18], and the second wave (October 2018 to November 2019) included 4,176 individuals of the wave 1 survey; at follow-up 5% (254) declined, 12% (595) had died, and < 1% (34) were not found, and response rate: 94%. HAALSI inclusion criteria were “permanent residence in the Agincourt health and socio-demographic surveillance system (HDSS) area during the 12 months prior to the 2013 HDSS census update round, and age 40 years or above on July 1, 2014, and mental capacity to consent to participation in this study, and exclusion criteria were participants who meet the inclusion criteria but do not wish to participate in this study.” [18] Participants responded to individual computer-assisted personal interviews (CAPI), and physical measurements [18].

The study protocol was approved by the “University of the Witwatersrand Human Research Ethics Committee (ref. M141159), the Harvard T.H. Chan School of Public Health, Office of Human Research Administration (ref. C13–1608–02), and the Mpumalanga Provincial Research and Ethics Committee.” Participants provided written informed consent. All methods were carried out in accordance with relevant guidelines and regulations.

### Measures

#### Dependent variable

Depressive symptoms (scores ≥ 3) were measured with the “Center for Epidemiological Studies-Depression Scale eight-item scale (CES-D 8) or the CES-D 20” [19, 20], internal consistency was 0.7 and 0.8 in wave 1 and 2, respectively.

#### Exposure variable

*Fruit and vegetable intake* was sourced from the following items:

1. “In a typical week, on how many days do you eat fruit? […vegetables?] (…days)” 2) “How many servings of fruit do you eat on a typical day? […vegetables] (on any one day) (…servings)” [use of show cards, serving size one standard serving = 80 g; fruits = ”1 medium size piece apple, banana, orange, etc.; ½ cup chopped, cooked, canned fruit, etc.; ½ cup fruit juice (juice from fruit, not artificially flavoured)”; vegetables = “1 cup raw green leafy vegetables (spinach, salad, etc.), ½ cup tomatoes, carrots, pumpkin, corn, Chinese cabbage, fresh beans, onion, etc., ½ cup vegetable juice”] [18].

#### Independent variables

Social and demographic data: household wealth, age, country of birth, education level, sex, and marital status [18].

Current tobacco use was defined as current smokeless and/or current tobacco smoking [18].

Alcohol dependence was assessed with the 4-item CAGE scale [21], (internal consistency 0.8).

Other dietary items included sugary drink consumption, “How many cans or bottles or cups of sugary drinks (e.g., Coke, Pepsi, Fanta, Sprite, etc.) do you drink in a typical week?” “[USE SHOWCARDS OF POPULAR DRINKS LOCALLY.]” [18].

Height and weight were measured, and Body Mass Index (BMI) calculated following World Health Organization (WHO) criteria (“underweight: < 18.5 kg/m^2^, normal weight: 18.5 to 24.9 kg/m^2^, overweight: 25 kg/m^2^ to 29.9 kg/m^2^, obesity: kg/m^2^ “) [22].

Hypertension was measured (averaging of the last two of three blood pressure readings) and defined based on National Committee criteria [23].

Dyslipidaemia: taking medication for high cholesterol and/or “total cholesterol > 6.21 mmol/L, HDL-C < 1.19 mmol/L, LDL-C > 4.1 mmol/L, triglycerides > 2.25 mmol/L.” [18].

Diabetes: taking glucose lowering medication and/or “fasting glucose (defined as > 8 h) > 7 mmol/L (126 mg/dL).” [18]

Physical activity (low, moderate, and high) was measured with the “General Physical Activity Questionnaire (GPAQ)” [24, 25].

### Statistical analysis

A total of 5,059 individuals (≥ 40 years) participated in wave 1, and of these 4,176 were followed at wave 2. Our analytic sample included participants who provided data on depressive symptoms at wave 2, giving a final sample of 3891. These sample restrictions were imposed as information of fruit and vegetable consumption and all other variables assessed in this analysis were obtained from wave 1. Descriptive statistics was used to describe baseline data. Chi-squared tests were used to test for differences in proportions. Univariable and multivariable logistic regression was applied to assess the association between fruit and vegetable intake at wave 1 and incident and persistent depressive symptoms. Incident depressive symptoms were assessed among those without depressive symptoms at wave 1 and referred to new cases of depressive symptoms at wave 2. Persistent depressive symptoms were measured only among those who had depressive symptoms at wave 1 and were defined as having depressive symptoms at wave 2. The multivariable models were adjusted for age, sex, education, wealth and marital status, alcohol dependence, current tobacco use, frequency of cans/bottles/cups of sugary drink consumption, physical activity, body mass index, hypertension, diabetes and dyslipidaemia. *p* < 0.05 was considered as significant. “Inverse probability weights were applied to account for attrition and mortality at follow-up” [26]. StataSE 15.0 (College Station, TX, USA) was used for statistical procedures.

## Results

### Sample characteristics

Baseline sample characteristics compared to fruit and vegetable consumption pattern are shown in Table 1. The mean age of the analytical sample (*N* = 3,891) was 61.0 years (SD = 12.4 years), and 43.9% were males. The prevalence of depressive symptoms at baseline was 15.5% (*N* = 598), and at wave 2 there were 835 new cases with depressive symptoms (incident depressive symptoms). Of those who had depressive symptoms at wave 1, 148 (30.8%) continued to have depressive symptoms at wave 2 (persistent depressive symptoms).

**Table 1:** Baseline sample characteristics Agincourt, South Africa

Baseline variables		Baseline sample	Fruit and vegetable servings/day			*p*-value^a^	Fruit servings/day			*p*-value^a^	Vegetable servings/day			*p*-value^a^
			0–1	2	≥ 3		0	1	≥ 2		0	1	≥ 2	
		*N* (%)	%	%	%		%	%	%		%	%	%	
All		3891	2452	881	526		933	2504	430		1286	2222	371	
Age (in years)	40–49 50–59 60–69 70–79 80 or more	742 (19.2) 1110 (28.7) 1018 (26.3) 681 (17.8) 313 (8.1)	68.2 64.9 62.3 58.7 63.9	20.9 22.6 23.9 25.0 20.6	11.0 12.5 13.8 16.3 15.5	0.017	22.9 23.5 23.6 23.7 32.5	66.0 65.1 65.6 65.4 56.6	11.1 11.4 10.8 10.8 10.9	0.093	39.7 34.6 30.7 28.3 32.7	54.5 57.1 59.0 58.9 55.1	5.8 8.3 10.4 12.8 12.2	< 0.001
Sex	Female Male	2184 (56.1) 1707 (43.9)	62.3 65.2	23.7 21.8	14.1 13.0	0.174	24.4 23.8	64.7 64.8	10.9 11.4	0.879	30.4 36.7	58.7 55.5	11.0 7.8	< 0.001
Education	None 1–7 years 8–11 12 or more	1681 (43.3) 1279 (32.9) 441 (11.4) 481 (12.4)	65.7 62.6 62.0 60.3	21.6 24.1 24.1 22.6	12.7 13.3 13.9 17.2	0.111	30.7 22.8 15.7 12.7	61.2 65.9 70.4 69.1	8.1 11.3 13.9 18.2	< 0.001	28.4 33.4 41.7 41.5	59.0 57.8 51.7 55.4	12.7 8.8 6.6 3.1	< 0.001
Marital status	Married/cohab Not married	2066 (53.1) 1823 (46.9)	61.8 65.5	24.4 21.1	13.8 13.4	0.035	20.6 28.2	67.8 61.2	11.6 10.6	< 0.001	31.9 34.6	59.4 54.9	8.7 10.5	0.013
Wealth index	Low Middle High	1531 (39.3) 763 (19.6) 1597 (41.0)	69.1 65.7 57.1	19.8 22.4 25.9	11.1 11.9 16.9	< 0.001	34.2 22.7 15.2	58.1 67.1 70.0	7.6 10.3 14.9	< 0.001	30.1 36.7 34.4	58.4 55.1 57.3	11.5 8.2 8.4	< 0.001
Alcohol dependence	No Yes	3838 (98.7) 52 (1.3)	63.3 76.9	23.1 5.8	13.6 17.3	0.013	23.8 48.1	65.1 42.3	11.1 9.6	< 0.001	33.2 30.8	57.3 57.7	9.5 11.5	0.858
Current tobacco use	No Yes	3316 (85.3) 572 (14.7)	62.9 67.1	23.3 20.2	13.8 12.6	0.152	22.6 33.1	65.7 58.9	11.7 8.0	< 0.001	33.1 33.5	57.9 53.7	9.0 12.8	0.012
Physical activity	Low Moderate High	1588 (41.1) 910 (23.5) 1368 (35.4)	60.1 65.7 65.8	24.5 21.9 21.7	15.4 12.5 12.5	0.009	20.2 21.4 30.4	66.7 68.5 60.0	13.1 10.1 9.5	< 0.001	30.7 35.1 34.4	59.6 57.4 54.7	9.6 7.5 10.8	0.007
Body mass index	Normal Under Overweight Obesity	1316 (35.2) 165 (4.4) 1091 (29.2) 1168 (31.2)	63.4 62.6 67.9 58.4	22.5 19.6 20.1 26.7	14.1 17.8 12.0 14.8	< 0.001	27.3 27.6 24.6 19.0	62.3 61.3 65.1 68.1	10.4 11.0 10.2 12.9	< 0.001	30.8 29.3 36.7 32.2	58.6 53.7 55.8 57.9	10.6 17.1 7.4 9.9	< 0.001
Hypertension	No Yes	1616 (42.2) 2215 (57.8)	66.9 60.7	20.3 24.8	12.7 14.5	< 0.001	26.5 22.4	62.4 66.4	11.1 11.1	0.014	35.6 30.8	56.4 58.4	8.1 10.8	< 0.001
Diabetes	No Yes	3225 (89.1) 395 (10.9)	63.7 60.5	22.9 24.1	13.4 15.4	0.405	24.9 18.2	64.4 68.9	10.7 12.9	0.011	32.9 31.1	57.6 58.7	9.5 10.1	0.754
Dyslipidaemia	No Yes	1881 (56.6) 1444 (43.4)	64.5 61.5	23.0 23.9	12.5 14.6	0.129	25.9 21.6	64.7 66.4	9.4 12.0	0.002	33.0 32.2	57.7 57.6	9.3 10.2	0.636
Cans/bottles/cups sugary drinks/week	0 1 2 ≥ 3	843 (21.8) 906 (23.4) 1106 (28.6) 1009 (26.1)	70.7 63.0 64.5 57.2	19.1 22.0 24.7 24.8	10.2 15.0 10.8 18.0	< 0.001	50.6 23.7 14.2 13.5	41.7 64.6 77.0 70.8	7.7 11.6 8.8 15.7	< 0.001	36.6 30.3 32.2 33.9	52.4 57.9 59.7 58.0	10.9 11.9 8.1 8.1	< 0.001
Depressive symptoms	No Yes	3268 (84.5) 598 (15.5)	63.2 65.1	23.2 21.2	13.6 13.7	0.570	23.5 27.1	65.1 63.2	11.3 9.7	0.133	33.4 32.0	57.4 56.9	9.3 11.1	0.367

^a^The difference in baseline characteristics by fruit and vegetable consumption pattern was tested using Pearson Chi-square statistics

Among those without depressive symptoms at wave 1, the prevalence of depressive symptoms at wave 2 (incident depressive symptoms) was higher among those with higher vegetable consumptions but did not differ with frequency of fruit consumption and frequency of fruit and vegetable consumption. Among those with depressive symptoms at wave 1, the prevalence of depressive symptoms at wave 2 (persistent depressive symptoms) did not differ with frequency of fruit and vegetable consumption, frequency of fruit consumption and frequency of vegetable consumption (see Table 2).

**Table 2:** Prevalence of incident or persistent depressive symptoms by fruit and vegetable consumption

Baseline variable	Incident depressive symptoms		Persistent depressive symptoms	
	%	*p*-value^a^	%	*p*-value^a^
*Fruit and vegetable servings/day*				
0–1	26.4	0.439	30.5	0.148
2	26.7		35.9	
≥ 3	28.9		24.5	
*Fruits servings/day*				
0	25.1	0.713	29.1	0.796
1	25.5		31.6	
≥ 2	27.2		30.1	
*Vegetable servings/day*				
0	26.0	0.009	32.8	0.307
1	24.5		28.8	
≥ 2	32.1		36.1	

^a^Pearson Chi-square statistics

Table 3 provides the association of fruit or vegetable consumption with incident and persistent depressive symptoms estimated by univariable and multivariable logistic regression. In univariable analysis, the consumption of two or more servings of vegetables/day was associated with a 1.34 (95% CI 1.02 to 1.77) times higher odds of incident depression. However, this became non-significant in multivariable analysis. There was no significant association between frequency of fruit consumption and incident depression, as well as between frequency of fruit consumption, vegetable consumption and persistent depressive symptoms (see Table 3).

**Table 3:** Univariable and multivariable associations of fruit and vegetable consumption and other covariates with incident or persistent depressive symptoms

Variable	Incident depressive symptoms		Persistent depressive symptoms	
	COR (95% CI)	AOR (95% CI)	COR (95% CI)	AOR (95% CI)
*Fruit servings/day*				
0 1 ≥ 2	1 (Reference) 1.03 (0.85 to 1.25) 1.10 (0.83 to 1.47)	1 (Reference) 1.11 (0.89 to 1.38) 1.20 (0.87 to 1.66)	1 (Reference) 1.13 (0.79 to 1.63) 1.04 (0.58 to 1.87)	1 (Reference) 1.50 (0.91 to 2.46) 1.30 (0.76 to 2.22)
*Vegetable servings/day*				
0 1 ≥ 2	1 (Reference) 0.91 (0.77 to 1.08) 1.34 (1.02 to 1.77)*	1 (Reference) 0.84 (0.71 to 1.01) 1.30 (0.97 to 1.73)	1 (Reference) 0.83 (0.59 to 1.17) 1.17 (0.69 to 1.97)	1 (Reference) 0.83 (0.54 to 1.26) 0.68 (0.36 to 1.27)
*Cans/bottles/cups sugary drinks/week*				
0 1 2 ≥ 3	1 (Reference) 1.12 (0.89 to 1.39) 1.18 (0.96 to 1.46) 1.12 (0.91 to 1.39)	1 (Reference) 1.06 (0.82 to 1.37) 1.12 (0.88 to 1.41) 1.18 (0.94 to 1.47)	1 (Reference) 0.82 (0.53 to 1.25) 0.71 (0.47 to 1.10) 0.91 (0.57 to 1.44)	1 (Reference) 0.73 (0.44 to 1.21) 0.69 (0.45 to 1.12) 0.66 (0.41 to 1.07)
Age (in years)	1.00 (0.99 to 1.01)	1.00 (0.99 to 1.01)	1.01 (0.99 to 1.02)	1.00 (0.98 to 1.01)
*Sex*				
Female Male	1 (Reference) 0.94 (0.81 to 1.09)	1 (Reference) 1.03 (0.84 to 1.26)	1 (Reference) 1.15 (0.84 to 1.57)	1 (Reference) 1.26 (0.81 to 1.96)
*Education*				
None 1–7 years 8–11 12 or more	1 (Reference) 0.91 (0.77 to 1.08) 0.83 (0.65 to 1.05) 0.89 (0.71 to 1.12)	1 (Reference) 0.89 (0.73 to 1.09) 0.88 (0.65 to 1.18) 0.95 (0.71 to 1.27)	1 (Reference) 0.68 (0.48 to 0.95)* 0.46 (0.24 to 0.87)* 0.49 (0.25 to 0.98)*	1 (Reference) 0.53 (0.35 to 0.82)** 0.39 (0.18 to 0.88)* 0.38 (0.15 to 0.89)*
Not married (base = Married/cohabiting	1.24 (1.04 to 1.44)**	1.20 (1.00 to 1.43)*	0.89 (0.69 to 1.21)	0.89 (0.59 to 1.34)
*Wealth index*				
Low Middle High	1 (Reference) 0.82 (0.67 to 1.01) 0.89 (0.76 to 1.05)	1 (Reference) 0.79 (0.63 to 0.99) 0.94 (0.77 to 1.14)	1 (Reference) 1.01(0.67 to 1.53) 0.94 (0.66 to 1.34)	1 (Reference) 1.05 (0.63 to 1.75) 1.20 (0.76 to 1.90)
Alcohol dependence	0.73 (0.37 to 1.45)	0.69 (0.32 to 1.45)	3.82 (1.15 to 12.65)	5.36 (1.24 to 23.30)*
Current tobacco use	1.31 (1.07 to 1.59)**	1.23 (0.97 to 1.56)	0.90 (0.59 to 1.36)	0.91 (0.51 to 1.63)
*Physical activity*				
Low Moderate High	1 (Reference) 0.97 (0.80 to 1.16) 0.98 (0.83 to 1.16)	1 (Reference) 0.95 (0.77 to 1.17) 0.93 (0.77 to 1.12)	1 (Reference) 0.63 (0.40 to 1.00)* 0.93 (0.65 to 1.31)	1 (Reference) 0.79 (0.46 to 1.36) 1.20 (0.78 to 1.84)
*Body mass index*				
Normal Underweight Overweight Obesity	1 (Reference) 1.25 (0.87 to 1.80) 0.92 (0.77 to 1.11) 1.07 (0.89 to 1.27)	1 (Reference) 1.10 (0.72 to 1.65) 1.00 (0.81 to 1.24) 1.11 (0.89 to 1.37)	1 (Reference) 1.24 (0.65 to 2.36) 0.81 (0.54 to 1.21) 0.85 (0.57 to 1.27)	1 (Reference) 1.20 (0.54 to 2.66) 0.80 (0.48 to 1.32) 0.99 (0.58 to 1.71)
Hypertension	0.96 (0.83 to 1.11)	0.93 (0.78 to 1.11)	1.27 (0.92 to 1.75)	1.06 (0.69 to 1.61)
Diabetes	1.17 (0.92 to 1.50)	1.06 (0.81 to 1.39)	1.17 (0.76 to 1.79)	1.30 (0.76 to 2.23)
Dyslipidaemia	1.14 (0.97 to 1.33)	1.15 (0.97 to 1.36)	1.02 (0.73 to 1.42)	0.82 (0.56 to 1.20)

*COR *Crude odds ratio, *AOR *Adjusted odds ratio, *p < 0.05, **p < 0.01, ***p < 0.001

## Discussion

In this first longitudinal study among an ageing population in Africa, we found no significant negative associations between fruit or vegetable intake and incident and persistent depressive symptoms four years later among middle-aged and older adults in South Africa. This was after adjusted for age, sex, education, marital status, wealth index, alcohol dependence, current tobacco use, physical activity, body mass index, hypertension, diabetes, dyslipidaemia, and sugary drinks intake. Non-significant longitudinal results were also found for fruit, vegetable, and fruit and vegetable consumption among middle-aged and older adults in Thailand [10], for fruit and vegetable consumption among older adults in Taiwan [11], for vegetable intake among older adults in Korea [9] and middle-aged women in Australia [13], and for fruit intake in older adults in Taiwan [12]. However, in four longitudinal studies among middle-aged and/or older adult populations, fruit and/or vegetable intake, and in two studies fruit intake [9, 13], and in one study vegetable intake [12]. Similar mixed results were found from a review of studies in adult populations [14], yet pooled estimates showed that fruit or vegetable intake reduced depressive symptoms [7].

Several possible explanations for the non-significant results in our study are here advanced. First, in our study the frequency of fruit and vegetable intake was very low, and it can be argued that larger quantities are needed to be able to show a protective effect against depression [17]. Second, we did not assess the frequency and diversity of intakes of specific categories of fruits and vegetables, which could be differently associated with depressive symptoms. For example, the types and/or low diversity of vegetables consumed in larger amounts may not contain the specific nutrients that have shown to be protective against depression [17]. Third, although we adjusted in our study for relevant confounders, including BMI, and physical activity, we did not adjust for other dietary factors (apart from soft drink intake), such as fish, nuts and red meat intake. Dietary consumption is complex, including various nutrients or types of food, making it hard to identify specific effects on depression [17]. Therefore, future studies should include the whole range of dietary behaviour in assessing its relationship with depression. Based on these findings further research is needed to address these limitations and include longer and more frequent follow-up assessments.

### Study strength and limitations

The strength of the study is the large sample size and longitudinal study design. Depressive symptoms were only assessed with a screening instrument. The measure of fruit and vegetable consumptions was limited since it did not assess the frequency of specific types of fruits and vegetable consumed, and cooking methods of the vegetables. We could not adjust for energy intake (except of sugary drinks intake), which could have influenced depressive symptoms. Furthermore, dietary behaviour, including health risk behaviours may have changed between wave 1 and wave 2.

## Conclusions

Fruit and/or vegetable intake was not significantly associated with incident and persistent depressive symptoms among middle-aged and older South Africans.

## Data Availability

“The data used in this study is publicly available at the Harvard Center for Population and Development Studies (HCPDS) program website (https://dataverse.harvard.edu/dataset.xhtml?persistentId=doi:10.7910/DVN/UELYSV).”
